# Experimentally determined relative biological effectiveness of cyclotron-based epithermal neutrons designed for clinical BNCT: *in vitro* study

**DOI:** 10.1093/jrr/rrad056

**Published:** 2023-08-22

**Authors:** Naonori Hu, Minoru Suzuki, Shin-ichiro Masunaga, Genro Kashino, Yuko Kinashi, Yi-Wen Chen, Yong Liu, Koki Uehara, Toshinori Mitsumoto, Hiroki Tanaka, Koji Ono

**Affiliations:** Particle Radiation Oncology Research Center, Industrial Equipment Division, Kyoto University, 2, Asashiro-Nishi, Kumatori-cho, Sennan-gun, Osaka 590-0494, Japan; Kansai BNCT Medical Center, Osaka Medical and Pharmaceutical University, 2-7 Daigaku-machi, Takatsuki-shi, Osaka 569-8686, Japan; Particle Radiation Oncology Research Center, Industrial Equipment Division, Kyoto University, 2, Asashiro-Nishi, Kumatori-cho, Sennan-gun, Osaka 590-0494, Japan; BNCT Research Center, Osaka Prefectural University, 1-1 Gakuen-cho, Naka-ku, Sakai, Osaka 599-8531, Japan; Advanced Medical Research Center, Nara Medical University, 840 Shijo-Cho, Kashihara, Nara 634-8521, Japan; Particle Radiation Oncology Research Center, Industrial Equipment Division, Kyoto University, 2, Asashiro-Nishi, Kumatori-cho, Sennan-gun, Osaka 590-0494, Japan; Department of Oncology, Taipei Veterans General Hospital, 201, Sec. 2, Shipai Rd., Beitou District, Taipei City, Taiwan 11217, Taiwan; Department of Radiation Oncology, Shanghai General Hospital, 100 Haining Road, Hongkou District, Shanghai 200080, China; Stella Pharma Corporation, ORIX Kouraibashi Building, 3-2-7 Kouraibashi, Chuo-ku, Osaka 541-0043, Japan; Industrial Equipment Division, Sumitomo Heavy Industries Ltd, 1-1, Osaki 2-chome, Shinagawa-ku, Tokyo 141-6025, Japan; Particle Radiation Oncology Research Center, Industrial Equipment Division, Kyoto University, 2, Asashiro-Nishi, Kumatori-cho, Sennan-gun, Osaka 590-0494, Japan; Kansai BNCT Medical Center, Osaka Medical and Pharmaceutical University, 2-7 Daigaku-machi, Takatsuki-shi, Osaka 569-8686, Japan

**Keywords:** BNCT, RBE, in vitro, fast neutron, accelerator

## Abstract

A neutron beam for boron neutron capture therapy (BNCT) of deep-seated tumours is designed to maintain a high flux of epithermal neutrons, while keeping the thermal and fast neutron component as low as possible. These neutrons (thermal and fast) have a high relative biological effectiveness in comparison with high energy photon beams used for conventional X-ray radiotherapy. In the past, neutrons for the purpose of BNCT were generated using nuclear reactors. However, there are various challenges that arise when installing a reactor in a hospital environment. From 2006, the Kyoto University Research Reactor Institute, in collaboration with Sumitomo Heavy Industries, began the development of an accelerator-based neutron source for clinical BNCT in a bid to overcome the shortcomings of a nuclear reactor-based neutron source. Following installation and beam performance testing, *in vitro* studies were performed to assess the biological effect of the neutron beam. Four different cell lines were prepared and irradiated using the accelerator-based neutron source. Following neutron and gamma ray irradiation, the survival curve for each cell line was calculated. The biological end point to determine the relative biological effectiveness (RBE) was set to 10% cell survival, and the D_10_ for each cell line was determined. The RBE of the accelerator-based neutron beam was evaluated to be 2.62.

## INTRODUCTION

Boron neutron capture therapy (BNCT) is a type of particle therapy which utilizes the high linear energy transfer (LET) particles that are emitted from the reaction of thermal neutrons with boron-10 atoms (^10^B(n,α)^7^Li). The emitted particles have a short range in tissue (few to several micrometres), thereby depositing almost all its energy in the cell where the reaction took place. Therefore, if sufficient quantities of boron-10 are accumulated in the tumour cells and very little accumulation occurs in the surrounding healthy tissue, BNCT becomes an ideal cancer therapy. The concept of BNCT was proposed back in 1936 by Locher [1], only 4 years after the discovery of the neutron by Chadwick. The majority of BNCT conducted worldwide utilized neutrons reactor neutrons. Between 1974 and 2014, the Kyoto University Reactor (KUR) was used to perform over 500 clinical BNCT irradiations at Kyoto University Research Reactor Institute (now known as Kyoto University Institute for Integrated Radiation and Nuclear Science (KURNS)) in Japan [2–5]. The new approach is to use an accelerator-based neutron system as an alternative because it has certain established benefits over a nuclear reactor. The system needs to be one that can be easily and safely implemented in a hospital for BNCT to advance as a routine treatment technique in cancer therapy.

Development of a cyclotron-based epithermal neutron source (CBENS) at KURNS began in 2006, which was a joint project between Kyoto University and Sumitomo Heavy Industries Ltd [6]. This system utilizes a 30 MeV proton beam with a beryllium target to generate neutrons. The neutrons traverse through a carefully designed beam shaping assembly (BSA) to reduce the energy down to the epithermal range (~10 keV), which has been shown to be the most suitable energy range for BNCT of deep-seated tumours [7]. The BSA is made of lead and iron, which are known as the moderator materials, and aluminium and calcium fluoride, which are known as the shaper materials. Further information on the BSA can be found elsewhere [8].

Before the system can be used to treat human beings, biological characterization of the neutron beam is necessary. The purpose of this paper is to report the relative biological effectiveness of the CBENS BNCT system and the methodology used to determine it.

## METHODS AND METHODS

### Cell-line

The cell-lines used in this study are summarized in Table 1. The SAS and U87-MG cell-lines were selected because their origins are from human tongue cancer and glioblastoma cell, respectively, which are the target areas for the planned clinical trial. The NB1RG cell-line was selected because the dose-prescribing organ for BNCT is usually the healthy skin. The SCCVII cell-line was selected because there is a track record of use in neutron irradiation experiments at KURNS. The cells were stored in a cryopreservation liquid CELLBANKER 1 (TOSC Ltd (formerly Jyuji Field Ltd, Japan)) at a temperature of −80 ± 2°C. The cultivation of the cells was performed using a CO_2_ incubator at a temperature of 37 ± 0.2°C with the carbon dioxide gas concentration set to 5 ± 0.2%. When the cell concentration reached 80% confluence, subculture was performed according to the following procedure.

a) The cells were washed using a 0.25% trypsin solution or trypsin–EDTA solution then trypsin solution was added.
b) The solution was processed in an incubator at 37°C.
c) Observation under a microscope was performed, and once the cells were almost detached from the bottom of the culture vessel, the growth medium was added.
d) The cells were suspended by pipetting and transferred to a centrifuge tube and centrifuged at 1000 rpm for 5 min. The supernatant was removed, and the growth medium was added, and the cells were seeded in a culture vessel.

**Table 1:** Summary of the cell-lines

Cell line	Supply source	Origin	Culture medium	Additive
SAS	RIKEN Cell bank	Human cell line derived from tongue cancer	RPM-1680	FBS with 10% concentration
SCCVII	Stanford University^a^	Murine squamous cell carcinoma	MEM	FBS with 12.5% concentration
U87-MG	American Type Culture Collection Cell Bank (ATCC)	Human glioblastoma cell line	MEM	FBS with 10% concentration
NB1RG	RIKEN Cell bank	Normal human skin fibroblast	α MEM	FBS with 10% concentration

^a^The cells were supplied by Prof Martin Brown from Stanford University, Department of Fundamental Radiation in Medicine in 1980. Later, in 1991, the strains were distributed and stored at KURNS.

### Preparation of the cell suspension

A minimum of 5 ml of the suspended cell was prepared and adjusted to have a concentration of 1 × 10^6^ cell/ml. To adjust the number of cells, 10–15 μl of a single cell suspension was placed on a haemocytometer, and the cells were manually counted under a microscope using a cell counting chamber. About 1 ml of the suspended cell was placed into a cryotube, and five samples were prepared, one being the control and the remaining four tubes for each dose level (2, 4, 6, 8 Gy).

### Irradiation

The ^60^Co source at KURNS (maximum activity of 414 TBq) was used for the gamma ray irradiation, and CBENS BNCT system was used for the neutron irradiation. The cell tubes were placed concentrically on a thin graph paper (thickness of 0.6 mm) and placed onto the 30 × 30 cm square field size of the CBENS irradiation system. The neutron beam was horizontal, and the tubes were placed perpendicular to the beam. A total absorbed dose of 2, 4, 6 and 8 Gy were delivered to each cell-line for both gamma and neutron irradiation.

To evaluate the neutron dose, the neutron spectrum corresponding to the position of the cryotubes was derived using the Monte Carlo simulation code MCNPX along with the ENDF/B-VII nuclear data library [9]. The free-in-air neutron and gamma ray dose rate at the surface of the lead layer (i.e. BSA exit) were calculated to be 3.41 and 1.34 Gy/h, respectively. Information on the neutron spectrum free-in-air can be found in Tanaka *et al*. [10]. The Kinetic Energy Released in Matter coefficient was used to evaluate the neutron dose at each position. A beryllium oxide thermoluminescence dosimeter (Panasonic UD-170LS) encapsulated in quartz glass was used for the measurement of gamma ray dose. The UD-170LS has some sensitivity to thermal neutrons, therefore a correction factor outlined in the reference paper was used to account for the thermal neutron contribution [11].

### Evaluation

The colony formation method was used to process the cells after irradiation. The incubation time for colony formation was 9–14 days after irradiation, depending on the cell-line. The staining protocol was crystal violet for the SAS and NB1RG, and 7.5 and 3% Giemsa station solution for the SCCVII and U87-MG, respectively. After colony counting, the survival curves were generated according to the linear quadratic model (LQ model). The biological end point to determine the RBE was set to 10% cell survival, and the corresponding dose (D_10_) was determined for both the gamma ray and neutron irradiation. The RBE of the pure neutron was determined using the expression below.


\begin{align*} \mathrm{\gamma} \!-\!\mathrm{dose}\ \left({\mathrm{D}}_{10}\right)\! \left(\mathrm{Gy}\right)&\!=\!\mathrm{neutron\!}-\mathrm{\!beam}\!-\mathrm{\!dose}\ \left({\mathrm{D}}_{10}\right)\! \left(\mathrm{Gy}\right)\!\times\! {\mathrm{RBE}}_{\mathrm{beam}} \\ &\!=\!\mathrm{pure}\ \mathrm{neutron}\ \mathrm{dose}\ \left({\mathrm{D}}_{10}\right)\! \left(\mathrm{Gy}\right)\!\times\! {\mathrm{RBE}}_{\mathrm{pure}\kern0.17em \mathrm{neutron}}\\&\qquad+\mathrm{contamination}\ \mathrm{\gamma} -\mathrm{dose}\ \left({\mathrm{D}}_{10}\right)\ \left(\mathrm{Gy}\right) \end{align*}

(1) \begin{align*} &{\mathrm{RBE}}_{\mathrm{pure}\kern0.17em \mathrm{neutron}}=\left(\mathrm{\gamma} -\mathrm{dose}\ \left(\mathrm{Gy}\right)\right.\nonumber\\&\qquad\left.-\mathrm{contamination}\ \mathrm{\gamma} \!-\!\mathrm{dose}\ \left(\!\mathrm{Gy}\right)\!\right)\!/\mathrm{pure}\ \mathrm{neutron}\ \mathrm{dose}\ \left(\!\mathrm{Gy}\!\right) \end{align*}

The neutron-beam-dose is equal to the pure neutron dose plus the contamination γ-dose, which is the dose resulting from the primary gamma ray component of the accelerator.

An alternative method to evaluate RBE is by eliminating the effect of the contamination gamma ray dose component by dividing the survival fraction of the neutron beam by the survival fraction of the contamination gamma ray. It was assumed that the interaction between pure neutrons and gamma rays would be independent, as in equation (2).


$$ {SF}_{n+\mathrm{\gamma}}={SF}_n\times{SF}_{\mathrm{\gamma}} $$

(2) \begin{equation*} {SF}_n=\frac{SF_{n+\mathrm{\gamma}}}{SF_{\mathrm{\gamma}}} \end{equation*}

where *SF_n_* is the survival fraction of the pure neutron.

Using this method, the dose survival points of the neutron beam were corrected to the dose survival points of the pure neutrons. Thereafter, dose survival curves were generated for each cell line. The D_10_ values were obtained from the corrected dose survival curve of the pure neutrons and compared with D_10_ of the γ-rays to determine the RBE.

### Comparison with reactor-based neutron source

As mentioned in the introduction, a large portion of the clinical BNCT irradiations were performed using the KUR Reactor. Therefore, it is worthwhile to compare the RBE of the neutrons generated by an accelerator with a reactor. Blue *et al*. proposed a method to obtain a relation for the absorbed dose-averaged neutron RBE versus neutron energy appropriate for BNCT using an empirical RBE-LET relationship [12].

The average total neutron RBE (RBE_T_) of both CBENS and KUR was calculated using the expression below.


(3) \begin{equation*} {RBE}_T=\frac{\int \varPhi \left({E}_n\right)\times{RBE}_T\left({E}_n\right)\ dE}{\int \varPhi \left({E}_n\right)\ dE} \end{equation*}

## RESULTS

### RBE determination

**Fig. 1:**
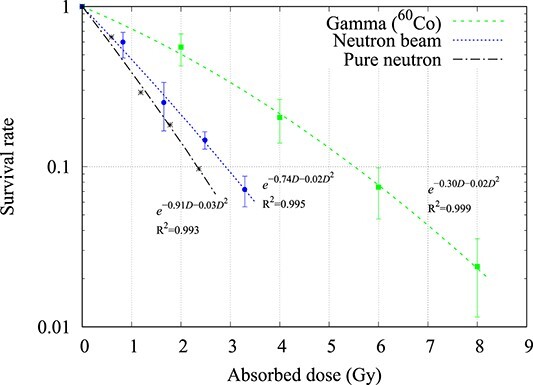
Cell survival curve of the SAS cell-line when irradiated with gamma rays (^60^Co) and neutrons (average value ± standard deviation, *n* = 2 or 3). The pure neutron survival curve was calculated from equation (2).

**Fig. 2:**
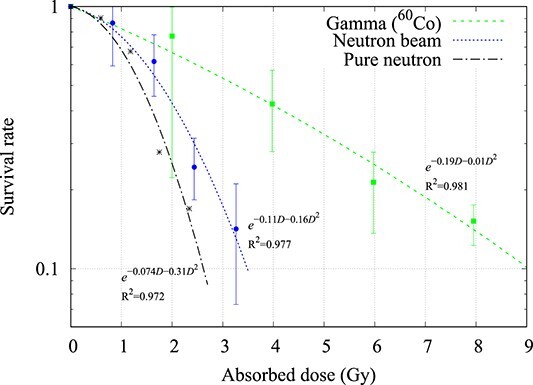
Cell survival curve of the SCCVII cell-line when irradiated with gamma rays (^60^Co) and neutrons (average value ± standard deviation, *n* = 2 or 3). The pure neutron survival curve was calculated from equation (2).

**Fig. 3:**
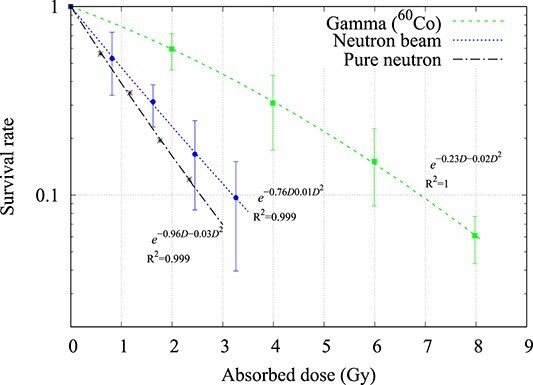
Cell survival curve of the U87-MG cell-line when irradiated with gamma rays (^60^Co) and neutrons (average value ± standard deviation, *n* = 2 or 3). The pure neutron survival curve was calculated from equation (2).

**Fig. 4:**
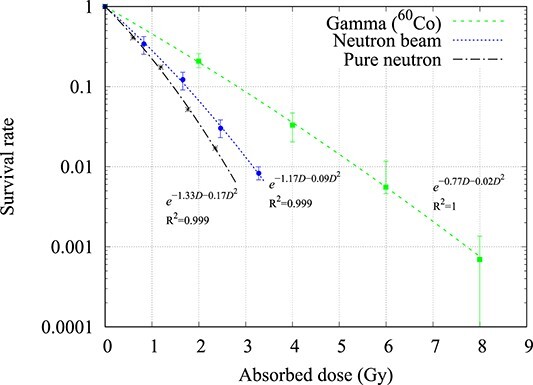
Cell survival curve of the NB1RG cell-line when irradiated with gamma rays (^60^Co) and neutrons (average value ± standard deviation, *n* = 2 or 3). The pure neutron survival curve was calculated from equation (2).

The survival rate of the SAS, SCCVII, U-87 and NB1RG cell-line after being irradiated by gamma rays and neutrons is shown in Figs 1–4, respectively. For the SAS cell-line, using the derived regression formula, when the natural log of the survival rate was 10%, the gamma ray dose was 5.50 Gy and the neutron beam (pure neutron + contamination gamma ray) dose was 2.90 Gy. The neutron beam includes the contamination gamma ray component (28.25%), so by subtracting this amount from 2.90 Gy, the dose from the pure neutron was calculated to be 2.08 Gy. The RBE_pure neutron_ was calculated to be 2.25 using equation (1) and 2.34 using equation (2).

For the SCCVII cell-line, the D_10_ value of the gamma ray dose was 9.05 Gy, and the neutron beam dose was 3.34 Gy. By subtracting the contamination gamma ray component, the dose from the pure neutron was calculated to be 2.40 Gy. The RBE_pure neutron_ was calculated to be 3.38 using equation (1) and 3.45 using equation (2).

For the U-87 cell-line, the D_10_ value of the gamma ray dose was 7.00 Gy, and the neutron beam dose was 3.15 Gy. By subtracting the contamination gamma ray component, the dose from the pure neutron was calculated to be 2.26 Gy. The RBE_pure neutron_ was calculated to be 2.70 using equation (1) and 2.75 using equation (2).

**Fig. 5:**
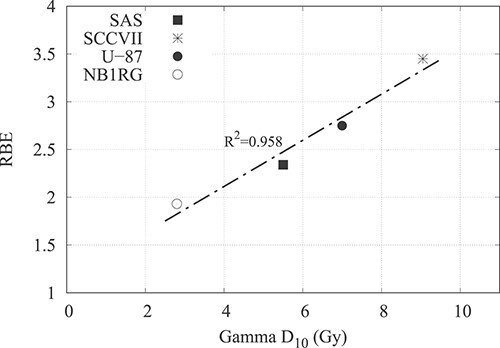
Plot showing the relation between the gamma D_10_ and the RBE of the neutron beam of the different cell lines.

For the NB1RG cell-line, the D_10_ value of the gamma ray dose was 2.80 Gy, and the neutron beam dose was 1.70 Gy. By subtracting the contamination gamma ray component, the dose from the pure neutron was calculated to be 1.22 Gy. The RBE_pure neutron_ was calculated to be 1.90 using equation (1) and 1.93 using equation (2). The relationship between the gamma D_10_ values and the evaluated RBE of the neutron beam for the different cell lines is shown in Fig. 5 and summarized in Table 2.

**Table 2:** The D_10_ values for each cell line under gamma irradiation (^60^Co) and neutron beam (contamination gamma + pure neutron), along with the evaluated RBE values for the pure neutron

Cell line	Gamma D_10_ [Gy ± SD]	Neutron beam D_10_ (Contamination gamma and pure neutron) [Gy ± SD]	RBE_pure neutron_	
			Equation (1)	Equation (2)
SAS	5.50 ± 0.7	2.90 ± 0.7 (0.82–2.08)	2.25	2.34
SCCVII	9.05 ± 4.1	3.34 ± 0.3 (0.94–2.40)	3.38	3.45
U87-MG	7.00 ± 1.8	3.15 ± 0.9 (0.89–2.26)	2.70	2.75
NB1RG	2.80 ± 0.3	1.70 ± 0.2 (0.48–1.22)	1.90	1.93

**Fig. 6:**
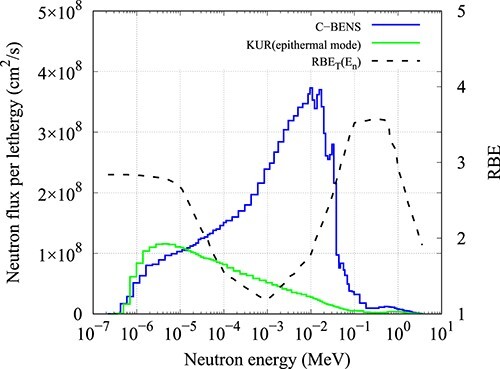
Plot of the total RBE for neutrons of energy *E_n_* derived by Blue *et al.* (RBE_T_(E_n_)) along with the neutron energy spectrum of CBENS and KUR.

The neutron energy spectrum of CBENS and KUR, along with the total RBE for neutrons of energy E_n_, is shown in Fig. 6. The RBE_T_ of CBENS and KUR epithermal mode was calculated to be 2.7 and 2.9, respectively.

## DISCUSSION

The survival fraction of the four different cell lines after neutron and gamma irradiation was experimentally determined and the evaluated RBE of the pure neutron ranged from1.90 to 3.45. Out of the four cell lines, the NB1RG cell-line had the highest sensitivity to gamma rays, resulting in the smallest RBE of 1.90–1.93. The calculated RBE using the two methods was found to be similar, within a percentage difference of <4% (1.55–3.85%).

The gamma ray and pure neutron D_10_ values for the four cell lines examined in this study showed a linear relationship (Fig. 5). This makes sense given that radiation sensitive cells have a low RBE, and radiation resistant cells have a high RBE. These characteristics of high LET radiation are well known, and the results are consistent with them. It is believed that this is because cells with low gamma ray sensitivity have a broad shoulder on their dose-survival curve, which has a significant impact on the cell survival in the low-dose region [13]. The size of the shoulder cannot be precisely stated in this study because the dose-survival curve was evaluated using the LQ model; however, there is a negative correlation between the size of the α value and the RBE. The RBE was evaluated at 10% survival rate as the dose at this level was approximately equal to the total neutron dose delivered to a healthy tissue for an exposure time of 1 h (~2 Gy) [14].

## Conclusion

The average neutron RBE of CBENS was determined to be 2.56 using equation (1) and 2.62 using equation (2). These values were similar to the RBE obtained using equation (3) (2.7), adding confidence to the experimentally evaluated value. Using the same equation, the RBE of the KUR epithermal neutron beam was slightly higher (2.9). This can be explained because KUR has a higher portion of lower neutrons than CBENS.

## Funding

This work was partly supported by the Japan Science and Technology Agency, Atomic Energy Strategic Basic and Fundamental Research Initiative.

## Conflict of interest

The authors declare that there are no conflicts of interest.

## Data availability

Data is owned by a third party. The data underlying this article were provided by Stella Pharma Coorporation and Sumitomo Heavy Industries, Ltd. under licence / by permission. Data may be shared on request to the corresponding author with permission of the above parties.
